# Capacity of health facilities for diagnosis and treatment of HIV/AIDS in Ethiopia

**DOI:** 10.1186/s12913-018-3347-8

**Published:** 2018-07-11

**Authors:** Amare Deribew, Sibhatu Biadgilign, Della Berhanu, Atkure Defar, Kebede Deribe, Ephrem Tekle, Kassahun Asheber, Tariku Dejene

**Affiliations:** 1 St. Paul Hospital Millennium Medical College, Addis Ababa, Ethiopia; 2 Nutrition International, Addis Ababa, Ethiopia; 3 Friedman School of Nutrition Science and Policy, Tufts University, Boston, USA; 4 London School of Hygiene and Tropical Medicine, London, UK; 5 Ethiopia Public Health Institute, Addis Ababa, Ethiopia; 6 Wellcome Trust Brighton & Sussex Centre for Global Health Research, Brighton & Sussex Medical School, Falmer, Brighton, UK; 7 Collage of Health Sciences, School of Public Health, Addis Ababa University, Addis Ababa, Ethiopia; 8 Federal Ministry of Health, Addis Ababa, Ethiopia; 9 Center for Population Studies, Addis Ababa University, Addis Ababa, Ethiopia

**Keywords:** Capacity, Health facilities, HIV/AIDS, Ethiopia

## Abstract

**Background:**

There are dearth of literature on the capacity of the health system to diagnose and treat HIV/AIDS in Ethiopia. In this study we evaluated the capacity of health facilities for HIV/AIDS care, its spatial distribution and variations by regions and zones in Ethiopia.

**Methods:**

We analyzed the Service Provision Assessment plus (SPA+) survey data that were collected in 2014 in all regions of Ethiopia. We assessed structural, process and overall capacity of the health system based on the Donabedian quality of care model. We included 5 structural and 8 process indicators and overall capacity score was constructed by taking the average of all indicators. Multiple linear regression was done using STATA 14 to assess the association of the location and types of health facilities with overall capacity score. Maps displaying the average capacity score at Zonal level were produced using ArcGIS Desktop v10.3 (Environmental Systems Research Institute Inc., Redlands CA, USA).

**Results:**

A total of 873 health facilities were included in the analysis. Less than 5% of the private facilities provided antiretroviral therapy (ART); had national ART guideline, baseline CD4 count or viral load and tuberculosis screening mechanisms. Nearly one-third of the health centers (34.9%) provided ART. Public hospitals have better capacity score (77.1%) than health centers (45.9%) and private health facilities (24.8%). The overall capacity score for urban facilities (57.1%) was higher than that of the rural (38.2%) health facilities (β = 15.4, 95% CI: 11.7, 19.2). Health centers (β = − 21.4, 95% CI: -25.4, − 17.4) and private health facilities (β = − 50.9, 95% CI: -54.8, − 47.1) had lower overall capacity score than hospitals. Facilities in Somali (β = − 13.8, 95% CI: -20.6, − 7.0) and SNNPR (β = − 5.0, 95% CI: -9.8, − 0.1) regions had lower overall capacity score than facilities in the Oromia region. Zones located in emerging regions such as Gambella and Benishangul Gumz and in remote areas of Oromia and SNNPR had lower capacity score in terms of process indicators.

**Conclusions:**

There is a significant geographical heterogeneity on the capacity of health facilities for HIV/AIDS care and treatment in Ethiopia. Targeted capacity improvement initiatives are recommended with focus on health centers and private health facilities, and emerging Regions and the rural and remote areas.

## Background

The world has seen a remarkable progress in the reduction of the burden of HIV/AIDS during Millennium Development Goals (MDG) period. However, HIV/AID continues to be a serious challenge during the Sustainable Development (SDG) era. In 2016, there were about 1.8 million new HIV infections, 36.7 million people living with HIV and 1 million HIV related deaths globally [1, 2]. Ethiopia has significantly expanded the HIV/AIDS interventions during the last 2 decades by decentralized the free Highly Active Antiretroviral Treatment (HAART) in the public facilities (health centers and hospitals) and private clinic and hospitals. However, the burden of HIV/AIDS is still high in the country. A total of 718,000 people were living with HIV in 2016 in Ethiopia [1, 3]. The incidence rate of HIV in Ethiopia in both males and females in 2016 was 40/100,000 which was higher than the incidence rate in 2010 (34/100,000) [Deribew A, Deribe K, Tessema GA, Adama YM: Burden of HIV/AIDS in Ethiopia from 1990 to 2016, submitted to Ethiopian Journal of Health Sciences]. The re-emergence of HIV/AIDS in Ethiopia could be due to low quality of care and inadequate coverage of high impact interventions. For instance, the 59% coverage HAART in Ethiopia is below the regional average and only 67% of Ethiopian know their HIV status that has become a hurdle for Ethiopia to achieve the 90–90-90 global targets [1].

Quality of care and capacity of the health facilities across the continuum of HIV/AIDS services play an essential role to improve treatment outcomes including adherence, viral suppression and mortality [4]. Recently, there is a growing need to increase the coverage, quality, and equity of services for HIV/AIDS in developing countries to provide longitudinal services and lifetime care for people living with HIV (PLWH) [5]. However, in sub-Saharan African countries adherence to standards of HIV/AIDS care at facilities is inadequate [6, 7]. A study conducted in Cape Town showed gaps in quality of care, positive prevention and missed opportunities for integrated care [8].

In Ethiopia, there were very few studies that assessed HIV/AIDS quality of care or capacity of health facilities for the diagnosis and treatment of HIV/AIDS. Most of the available studies included few health facilities in limited geographic areas and their major focus were perceived quality of care and factors associated with client satisfaction [9–12].

In this study we assessed the capacity of the health system for HIV/AIDS care in Ethiopia using the Donabedian quality of care framework and that included structural, process indicators and overall capacity score [13]. The study is the first of its kind to assess the capacity of the health facilities for HIV/AIDS care comprehensively and capacity variation at Zone level in Ethiopia. The policy of the Ethiopian government to ensure a decentralized and high quality HIV/AIDS services at the district level both in the private and public facilities. The study may help the government of Ethiopia and development partners to tailor their interventions based on the heterogeneity of the capacities of the health facilities in Ethiopia. The study could also help other countries to design similar studies to improve the capacity of the health facilities and quality of HIV/AIDS care and treatment.

## Methods

Ethiopia uses a three-tier health system. The primary health care units (composed of a health center and five satellite health posts) are linked to the district hospitals to provide basic and advanced services for HIV/AIDS and other major infectious diseases. HIV counselling and Testing (HCT), ART and prevention of mother-to-child transmission of HIV (PMTCT) are provided at the hospitals and health centers. The tertiary hospitals serve as a referral sites for the district hospitals [14]. The current study used data from the Service Provision Assessment plus (SPA+) survey. Briefly, SPA+ was conducted in 2014 in all the nine regional states of Ethiopia: Tigray, Afar, Amhara, Oromia, Somali, Benishangul-Gumuz, SNNP, Gambella, Harari and two city administrations, Addis Ababa and Dire Dawa [15]. Regions such as Somali, Afar, Gambella and Benishangul-Gumuz are referred as emerging regions based on their lower level of development and remote locations compared to the other regions in Ethiopia. The SPA+ was a cross-sectional survey of representative health facilities conducted using a sample of the formal sector health facilities in Ethiopia. Stratified sampling was utilized to select representative health facilities according to their type. A comprehensive list of health facilities by type was obtained from the Federal Ministry of Health to guide the sampling. Overall, SPA included 1327 health facilities namely 223 hospitals, 298 health centers, 321 health posts and 485 private facilities [16]. In the current analysis, health posts, pharmacies and individual doctors’ offices were not included since they did not provide HIV/AIDS care and treatment services. We included all relevant health facilities that provided HIV/AIDS services and hence the sample size is similar to the original SPA survey.

The SPA+ collected data on six major thematic areas including health infrastructure; facility audit questionnaire focusing on availability of trained personnel, clinical and laboratory services, medicines, and guidelines; observations and clinical knowledge assessment by interviewing health workers based on vignettes/cases. The SPA survey instruments were pretested and adopted from MEASURE DHS project, and Service Delivery Indicator (SDI) tools [16].

**Fig. 1:**
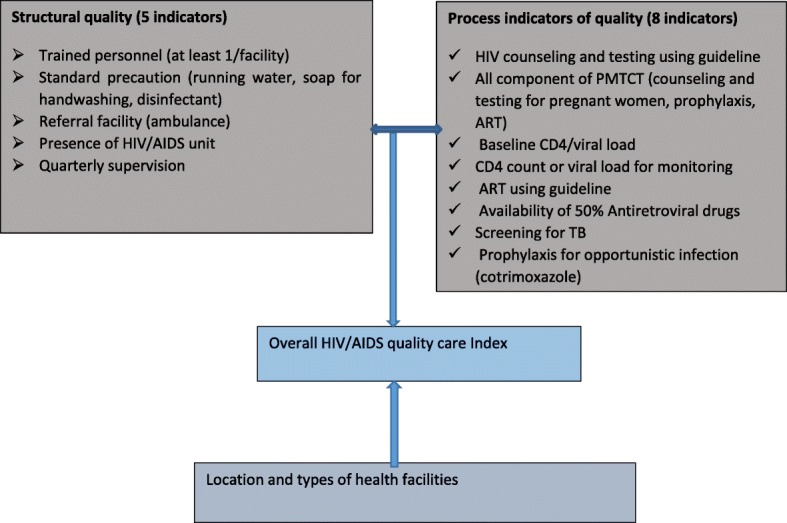
Conceptual framework that shows factors affecting overall HIV/AIDS quality of care index

We assessed structural, process and overall capacity based on the Donabedian quality of care framework [17, 18]. On the other hand, the indicators in each dimensions were in line with the WHO recommended HIV quality of care indicators [19]. In this paper we prefer to use the term capacity score rather than quality of care since we didn’t observe the practice of health workers for HIV/AIDS services. The data collection was mainly based on self-report and observation of availability of guidelines, supplies and spaces. We included 5 structural and 8 process capacity indicators as shown in Fig. 1. A score of 1(presence) or 0(absence) was given for each indicator. For instance, standard precaution was assessed based on the availability of all of the relevant indicators including running water, soap for handwashing and disinfectants. On the other hand, availability of PMTCT service was given score of 1 if the health facility had all of the relevant indicators including counseling and testing for pregnant women, prophylaxis and ART (Fig. 1). The final capacity score on structure and process dimension and the overall capacity score was constructed by taking the average of all indicators and rescaling it to 100.

The SPA+ data were cleaned and descriptive and multivariate analyses were done using Stata 14 (Version 14.0, Stata Corp LP, College Station, Texas, USA). Capacity score index was a continuous outcome variable that fulfilled the normality assumption. The major exposure variables included types of health facility, regions and location of health facilities (urban vs. rural). Bivariate analysis was done to see the association between each exposure variable with the outcome variable. Exposure variable that showed significant association with the outcome variable (*P* < 0.05) were included in the final model using stepwise Multipole Linear Regression. Maps displaying the average quality score at Zonal level were produced using ArcGIS Desktop v10.3 (Environmental Systems Research Institute Inc., Redlands CA, USA).

## Results

A total of 873 health facilities were included in the analysis. 32.6, 53.7 and 74.5% of these health facilities had ART, PMTCT and HCT services respectively. HCT was provided by only 45% of the private health facilities. Larger proportion of hospitals (98%) and health centers (94%) provided HCT. Less than 50 % of health facilities provided HCT in some of the emerging regions such as Gambella (45%) and Benishangul Gumuz (44.4%). Smaller proportion of the private health facilities (12.5%) had PMTCT services. Few number of health facilities in the emerging regions such as Gambella (25%), Benishangul Gumuz (33%) and Harari (37%) provided PMTCT services. About one third of the health centers (32.9%) and very few private facilities (0.8%) provided antiretroviral therapy (ART). ART was available in few number of health facilities in the rural areas (14.3%) and some of the regions such as Somali (17%), Gambella (17.5%), SNNPR (23.3%), and Harari (26%) (Table 1).

**Table 1:** HIV/AIDS care and services by types of health facilities

Facility characteristics	HIV counselling and Testing	PMTCT	Antiretroviral therapy	# Facilities with completed interviews [Unweighted]
	n(%)	n (%)	n (%)	
Types of health facility				
Health centers	275(94.2)	224(76.7)	96(32.9)	292
Hospitals	210(98.1)	199(93)	186(86.9)	214
Private facilities	165(45)	46(12.5)	3(0.8)	367
Location of health facilities				
Urban	422(78.4)	301(55.9)	237(44.1)	538
Rural	228(68.1)	168(50.1)	48(14.3)	335
Regions				
Tigray	80(87.9)	63(69.2)	40(44)	91
Afar	37(88.1)	17(40.5)	12(28.6)	42
Amhara	98(76.6)	84(65.6)	40(31.3)	128
Oromia	115(70.1)	88(53.7)	65(39.6)	164
Somali	36(76.6)	23(48.9)	8(17)	47
Benishangul Gumz	16(44.4)	12(33.3)	9(25)	36
SNNP	102(79.1)	77(59.7)	30(23.3)	129
Gambella	18(45)	10(25)	7(17.5)	40
Harari	27(77.1)	13(37.1)	9(25.7)	35
Addis Ababa	82(73.9)	60(54.1)	52(46.8)	111
Dire Dawa	39(78)	22(44)	13	50
Total	650(74.5)	469(53.7)	285	873

HIV/AIDS care and treatment capacity was assessed among facilities that had one or more HIV/AIDS services. Few health centers (17.8%) and private health facilities (24.9%) had the standard precautions. Only one third of the health centers and a quarter of the private health facilities provided HCT services using national guidelines. Less than 5% of the private facilities provided ART using national guideline and had baseline CD4 count or viral load and tuberculosis screening mechanisms. Nearly a quarter of the health centers (28.4%) provided ART using national guideline. At least 50% of antiretroviral drugs, baseline CD4 count or viral load, CD4 count/viral load for monitoring and TB screening were available in 3.6, 26.2, 22.9, and 25.1% of the health centers respectively. The overall quality index score was lower for private health facilities (mean/SE = 26.5/1.1) than that of the health centers (mean/SE = 46.7/1.3) and hospitals (mean/SE = 76.2/1.2) (Table 2).

**Table 2:** HIV/AIDS Quality of care dimensions by types of health facilities

Capacity Indicators	Proportion of health facilities with quality indicators		
	Health centers	Public hospitals	Private facilities
Structural Indicators	n(%)	n(%)	n(%)
Availability of trained personnel	193(70.2)	201(95.7)	79(46.7)
Standard precautions	49(17.8)	92(43.8)	42(24.9)
Referral capacity (e.g ambulance)	263(95.6)	198(94.3)	85(50.3)
Presence of Separate room for HIV/AIDS care	253(92)	193(91.9)	132(78.1)
Quarterly supervision from region or federal	209(76)	199(94.8)	115(68)
Process indicators			
*HIV counseling and Testing (HCT)* using guideline	91(33.1)	143(68.1)	41(24.3)
*All components of PMTCT service*	223(81.1)	199(94.8)	46(27.2)
*Baseline CD4 count* or viral load before treatment	72(26.2)	167(79.5)	3(1.8)
Antiretroviral therapy(ART) using national guideline	78(28.4)	158(75.2)	2(1.2)
*CD4 count* or viral load for monitoring	63(22.9)	149(71)	3(1.8)
Availability of Antiretroviral drugs	10(3.6)	69(32.9)	0(0)
Screening for Tuberculosis	69(25.1)	136(64.8)	7(4.1)
Preventive Treatment for opportunistic infection (cotrimoxazole)	97(35.3)	176(83.8)	28(16.6)
Quality score	*Mean(SE)*	*Mean(SE)*	*Mean(SE)*
	46.7(1.3)	76.2(1.2)	26.5(1.1)
Total number of facilities (Unweighted)	275	210	169

The overall capacity score for urban facilities was higher than that of the rural health facilities (β = 14.2, 95% CI: 10.6, 17.8). Health centers (β = − 20.4, 95% CI: -24.2, − 16.5) and private health facilities (β = − 48.4, 95% CI: -52.1, − 44.8) had lower overall capacity score than hospitals. Facilities in Somali (β = − 12.9, 95% CI: -19.4, − 6.4) and SNNPR (β = − 4.7, 95% CI: -9.4, 0.0) regions had lower overall capacity score than facilities in the Oromia region. However, the overall capacity score in Dire Dawa (β = 7.7, 95% CI: 1.2, 14.2) was higher than that of the Oromia region (Table 3).

**Table 3:** Determinants of HIV/AIDS quality of care

Variables	Mean	SE	Crude Model			Adjusted Model		
			Beta	95% CI		Beta	95% CI	
Residence				LL	UL		LL	UL
Urban	57.1	1.3	17.6	13.5	21.7	*14.2(P<0.05)*	*10.6(P<0.05)*	*17.8(P<0.05)*
Rural *[Ref]*	39.6	1.4						
Types of Facility								
Health Center	46.7	1.3	−29.5	−32.9	−26.1	*−20.4(P<0.05)*	*−24.2(P<0.05)*	*−16.5(P<0.05)*
Private facilities	26.5	1.1	−49.7	−53.5	−45.8	*−48.4(P<0.05)*	−*52.1(P<0.05)*	*−44.8(P<0.05)*
Hospital *[Ref]*	76.2	1.2						
Region								
Oromia *[Ref]*	57.4	2.8						
Tigray	59.8	2.7	2.4	−5.0	9.8	3.8	−1.2	8.8
Afar	39.5	3.7	−17.9	−27.5	−8.3	−5.5	−12.0	1.0
Amara	48.9	2.8	−8.5	−15.4	−1.5	−0.4	−5.1	4.3
Somali	39.1	3.6	−18.3	−28.0	−8.6	−12.9	− 19.4	−6.4
Benishangul Gumuz	56.2	5.6	−1.1	−14.7	12.4	1.3	−7.9	10.6
SNNP	41.8	2.5	−15.6	−22.5	−8.7	*−4.7(P<0.01)*	*−9.4(P<0.05)*	*0.0(P<0.05)*
Gambella	40.2	5.6	−17.2	−30.1	− 4.4	−6.1	− 14.8	2.6
Harari	43.3	5.4	−14.1	−24.9	− 3.3	0.3	−7.2	7.8
Addis Ababa	60.9	2.7	3.5	−3.8	10.8	−0.1	−5.2	5.0
Dire Dawa	52.7	3.6	−4.7	−14.1	4.7	*7.7(P<0.05)*	*1.2(P<0.05)*	*14.2(P<0.05)*

**Fig. 2:**
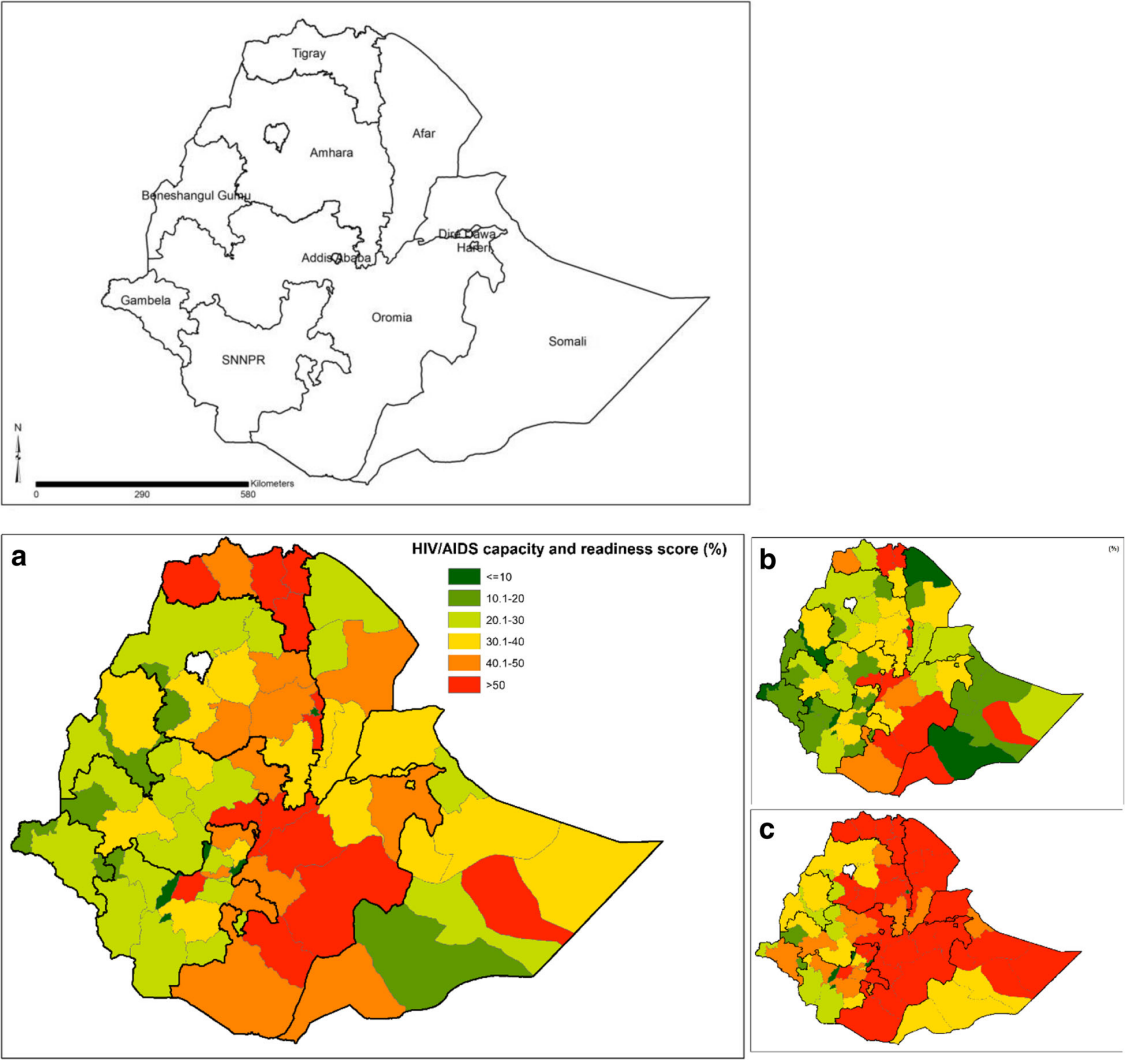
**a**) Overall **b**) Process and **c**) structural HIV services capacity and variation by Zones in Ethiopia. The regional states of Ethiopia are shown at the top

Spatial analysis showed that majority of the Zones in Ethiopia had similar capacity scores in terms of structural quality. However, HIV/AIDS capacity score was poor in the southwest, northeast and southeast part of Ethiopia. Most of the Zones with poor capacity score are located in emerging regions such as Afar, Gambella and Benishangul Gumuz and in remote areas of Oromia and SNNPR regions (Fig. 2).

## Discussion

The present study has provided a comprehensive evidence on the variations of HIV/AIDS capacity score by locations and types of health facilities at national level. HIV/AIDS capacity score is poor in rural areas, private health facilities and in remote zones of emerging regions in Ethiopia. Less than 5% of the private facilities has provided ART and used CD4 count or viral load for monitoring. On the other hand, only a third of the health facilities have provided HCT and ART services using national guidelines.

The low coverage and health facilities’ capacity for HIV/AIDS in rural and remote Zones of Ethiopia could be a hurdle to achieve the 90–90-90 global targets of HIV/AIDS by 2020 [1]. Based on these global targets, 90% of the population should know their HIV status; 90% of those who know their HIV should be treated and 90% of the treated should be virally suppressed by 2020. HCT by a trained personnel is a powerful intervention to achieve the first target (90% of the population should know their HIV status) and link HIV positive individuals to further treatment and care [20]. In the present study, only one-third of the health centers and a quarter of the private facilities have HCT services using national guidelines. Lack of trained staff, poor counseling skills and low awareness of clients could contribute for the poor quality of HCT service. A study conducted in Addis Ababa shows that absence of counsellors, poor counseling and poor awareness of clients on HCT were the major barriers for the PMTCT services [21]. Similarly, Asefa and colleagues have reported that lack of training and feedback on job performance and inadequate pay were the main challenges of the PMTCT service providers [22]. A multi-country study in Kenya, Uganda and Burkina Faso reveals that service providers face several challenges such as resource constraint, staff shortage, high workload and inadequate infrastructure to carryout HCT [23]. In our study the private facilities have poor quality of care in terms of HCT than the public health facilities which is contrary to a study in Zambia that showed better quality of care in the private facilities than the public sector [24].

Our findings show that more than 50% of private facilities and about a third of the health centers do not have trained personnel for HIV/AIDS related services. However, majority of the hospitals (96%) have at least one trained personnel which is in line with a study done in Northwest Ethiopia [9]. Literature shows that HIV/AIDS care and treatment services by trained personnel is associated with better survival, viral suppression, and low rate of treatment failure [25]. Essential laboratory services such as baseline and regular CD4 counts and viral load are very essential to monitor the effectiveness of ART. In the current study, more than 70% of hospitals use baseline or monitoring CD4 counts or viral load which is higher than the findings of a study in Uganda that shows only 37% of hospitals have basic laboratory including CD4 counts [6]. However, majority of the health centers and private facilities in the current study lack basic laboratory for HIV/AIDS that needs attention during the SDG era. Cotrimoxazole prophylaxis has been proven in reducing mortality in people living with HIV/AIDS [26]. Majority of the hospitals (80%) in the current study use cotrimoxazole prophylaxis compared to the low proportion of health centers (35%) and private clinics (17%) that uses cotrimoxazole prophylaxis.

The poor health facilities capacity in the rural, remote zones and private facilities could be explained by several factors. First, the number of trained personnel in rural areas, private sectors and remote zones could be inadequate to provide HIV related services. Second, HIV/AIDS resources and materials including laboratory facilities could not be distributed equitably in these areas. Third, the demand and health service utilization for HIV/AIDS in remote locations could be affected by poor health seeking behavior and stigma. A study in Jimma shows that stigma and prejudice poses serious challenge for the TB/HIV programs [27].

The study has several strengths. First, the study includes all regions in Ethiopia and showed HIV/AIDS capacity score variation by zones for informed decision making. Second, we have included most of the internationally recommended indicators of capacity of HIV/AIDs treatment and care [28]. However, it has some limitations. The study used secondary data based on interview of health workers and facility audit. There were no observations of the services of HIV/AIDS including HCT, PMTCT and ART provision. Second, this study didn’t assess quality of care on the perspectives of the clients and client-provider interactions were not assessed. Lastly, the cross sectional nature of the study could not allow us to assess trends the performance of the health facilities.

Our findings clearly showed that there are clear inequalities in capacity of health facilities for HIV/AIDS treatment and care. Indeed, they are a clear reminder that national averages do not tell the full story. The maps highlight stark heterogeneities. Zones, in Benishangul Gumuz, Gambella and Western Oromia and SNNP have lower HIV/AIDS health system capacity. To achieve the national and global 90–90-90 targets of HIV/AIDS by 2020, it is crucial to address HIV/AIDS health system capacity. Our analysis here would serve a purpose in responsibility to act, we have clearly shown where to direct resource and improve quality of services.

## Conclusions

The capacity of health facilities for HIV/AIDS care and treatment is poor in the private facilities and health centers compared to hospitals. Capacity score of health facilities for HIV/AIDS declines as we go to the remote zones in emerging regions. Future HIV/AIDS interventions should focus on the private sectors and the rural and remote areas to ensure equity and high quality of care.

## Abbreviations

ART: Antiretroviral treatment
DHS: Demographic and Health Survey
HAART: Highly active antiretroviral treatment
HCT: HIV counseling and Testing
HIV/AIDS: Human immunodeficiency Virus/Acquired immunodeficiency Syndrome
MDG: Millennium Development Goals
PLWH: People living with HIV
PMTCT: Prevention of Mother-to-child Transmission of HIV
SDG: Sustainable Development Goals
SNNPR: Southern Nation and Nationalities Peoples Region
SPA+: Service Provision Assessment Plus
WHO: World Health Organization
